# Why do Socioeconomic Differences in Women’s Living Standards Converge After Union Dissolution?

**DOI:** 10.1007/s10680-022-09620-9

**Published:** 2022-06-07

**Authors:** Bram Hogendoorn

**Affiliations:** 1 University of Amsterdam, Nieuwe Achtergracht 166, 1018 WV Amsterdam, The Netherlands; 2 Netherlands Interdisciplinary Demographic Institute, Lange Houtstraat 19, 2511 CV The Hague, The Netherlands

**Keywords:** Divorce, Financial independence, Income inequality, Matching, Difference-in-differences, Decomposition

## Abstract

Union dissolution is a critical event for women’s living standards. Previous work has found that women in high-income unions lose more from union dissolution than women in low-income unions. This study proposes two mechanisms to explain this “convergence” in living standards. The compensation mechanism concerns the ability to compensate the loss of partner earnings with alternative sources of income, whereas the partner independence mechanism concerns how much women stand to lose from dissolution in the first place. To test these mechanisms, the author drew on a unique administrative dataset from the Netherlands, covering women who experienced dissolution within ten years after union formation (*N* = 57,960). A decomposition analysis showed that convergence was not driven by compensation: women from all income groups decreased their household size and re-partnered, women from low-income unions increased transfer income, and women from high-income unions increased personal earnings and decreased tax payments. Instead, convergence was driven by partner independence: women from lower-income unions depended relatively less on their partners because they relied more on transfer income prior to dissolution. These results demonstrate how partners’ interdependence moderates the consequences of life events. The welfare state plays a crucial role in this process.

## Introduction

Romantic unions have become increasingly unstable during the past half a century, and more than a third of all unions today end in separation or divorce (Kalmijn & Leopold, 2021; Perelli‐Harris et al., 2017). Union dissolution has large financial consequences for women. Women lose 7% to 44% of their household income following union dissolution (Andreß et al., 2006; Bonnet et al., 2021; De Vaus et al., 2017; Holden & Smock, 1991; Uunk, 2004) and these losses often persist for several years (Leopold, 2018). Especially compared to men, who experience little change, union dissolution is a critical event for women’s standard of living.

Concerns about union dissolution have focused on women in low-income unions. Women in low-income unions appear more vulnerable to the consequences of dissolution than women in high-income unions, because they are less often dual earners, tend to assume a larger share of housework and childcare, and are more likely to become the sole custodial parent after dissolution (Cancian et al., 2014; Harkness, 2013; Mansour & McKinnish, 2014). While they might benefit from increases in public transfers, these increases are often limited (Bonnet et al., 2021; Tach & Eads, 2015). Therefore, theory predicts that socioeconomic differences in women’s living standards widen following union dissolution.

Surprisingly, however, a vast literature documents that income losses related to union dissolution are concentrated in women from high-income unions (Bonnet et al., 2021; Brewer & Nandi, 2014; Duncan & Hoffman, 1985; Fisher & Low, 2016; Jarvis & Jenkins, 1999; Rowe & Lown, 1990; Uunk, 2004; Weiss, 1984; Weitzman, 1981). Whereas women from high-income unions witness steep declines in their standard of living, women from low-income unions witness little change or even improve their situation somewhat. Socioeconomic differences in women’s living standards thus seem to narrow following dissolution, a phenomenon I will refer to as “convergence.”

These findings raise questions about the mechanism by which women’s living standards converge. Previous studies have pointed to a compensation mechanism, whereby women compensate the loss of partner earnings by reemployment, repartnering, increased tax-benefit income, and favorable custody arrangements (Bonnet et al., 2021; Fisher & Low, 2016; Jarvis & Jenkins, 1999; Tach & Eads, 2015). If women from low-income unions have a compensatory advantage over women from high-income unions, this might explain the convergence in living standards after dissolution. In this study, however, I argue that another mechanism is at work. The partner independence mechanism applies when, prior to dissolution, women’s financial standing depends relatively little on their partner because partner earnings make up a small portion of the total household income (Lister, 1994; Oppenheimer, 1997). If women in low-income unions are more independent of their partner than women in high-income unions, they stand to lose less upon leaving that partner, which would explain the convergence in living standards after dissolution. Neither of these mechanisms have been formally explored to date, leaving the question of convergence unanswered.

Hence, this study examines the convergence in women’s living standards following union dissolution. I draw on longitudinal register data from the Netherlands to follow women in coresidential unions that started between 2003 and 2005 for ten years. Matching separating women (*N* = 57,960) to partnered women, I apply a difference-in-differences design to estimate the dissolution penalty in each pre-dissolution income group. I then decompose the dissolution penalty into changes in household composition, ex-partner earnings, personal earnings, new partner earnings, transfer income, and tax payments, and show how these changes give rise to compensation and partner independence.

The results contribute to the field in several ways. First, they give greater insight into socioeconomic differentials in the dissolution penalty. Previous research has shown that the dissolution penalty differs by income group but has paid little attention to the income sources underlying these differences. Using population-wide register data, I provide a precise comparison between income groups and their sources of income. Furthermore, the results demonstrate the dual role of social policy, which affects both the ways in which women compensate the loss of partner earnings and the extent to which women financially depend on their partner. The Netherlands poses an interesting context here because its policies are relatively generous to low-income households (Wang & Van Vliet, 2016). Finally, this study presents the compensation and partner independence mechanisms for the event of union dissolution. These mechanisms easily extend to other events, aiding wider debates on demography and social stratification.

## Theoretical Expectations

The notion that union dissolution results in socioeconomic convergence can be traced to the work of Biblarz and Raftery (1993). Their study of occupational mobility showed that family dissolution increased the resemblance between sons of different backgrounds, because sons of high-status fathers were more likely than sons of low-status fathers to experience downward occupational mobility if they grew up in a non-intact family. Similar forms of convergence have been found for children’s education and earnings following parental union dissolution (Bernardi & Boertien, 2016; Bratberg et al., 2014).

Although previous work has been concerned more with child than with adult outcomes, it draws attention to two mechanisms relevant for adults. The first mechanism regards compensation (cf. Bernardi, 2014). Compensation may be achieved by reducing household size, expanding earnings from labor, adding new earners to the household, increasing transfer income, or lowering tax payments. This mechanism has been central in the literature and, consequently, much work has focused on child custody, reemployment, repartnering, and changes in tax-benefit income. The second mechanism is what I name “partner independence.” Partner independence entails a situation in which women’s financial standing is relatively independent of that of their partner, so that women lose little when splitting from that partner. This may be achieved because women earn similarly to (or out-earn) their partner or because they rely on alternative sources of income, notably public transfers. This mechanism has received attention in literature on the antecedents of union dissolution (for review, see Oppenheimer, 1997; Rogers, 2004), but less so in literature on the consequences of dissolution. As I lay out below, this is unfortunate, since the mechanism brings into focus how partners’ interdependence during their union shapes the consequences of dissolution as well as the role of the welfare state therein.

The direction and strength of these mechanisms drive the degree to which women’s living standards converge following union dissolution. Arguments on compensation paint a mixed picture. On the one hand, women from lower-income unions are closer to the income thresholds for receiving welfare, so they are better positioned for replacing income losses with welfare transfers (Bonnet et al., 2021). On the other hand, women from higher-income unions tend to be higher educated, more active in the labor force, and better able to increase their working hours (Jansen et al., 2009; Tamborini et al., 2015), so it may be easier for them to increase their earnings. Women in higher-income unions also pay a higher tax rate, so they benefit from the change to a lower tax bracket when splitting from their partner (Avram et al., 2014). Finally, changes in household composition probably benefit women across all income groups. While women in lower-income unions have larger families before dissolution (Balbo et al., 2013) and therefore more scope to reduce the number of household members they share their income with, they are less likely to share child custody with their ex-partner (Cancian et al., 2014), so that the actual reduction in household size may differ little. Repartnering probably also benefits women across all income groups, since both the probability and the consequences of repartnering appear unrelated to pre-dissolution household income (Fisher & Low, 2016; Shafer & James, 2013). Together, these arguments suggest that the compensation mechanism operates in countervailing directions, making it an unlikely candidate for explaining the convergence in living standards following union dissolution.

Instead, partner independence may be the dominant mechanism to explain the convergence in women’s living standards. The main argument is that lower-income unions rely relatively less on earnings, since they supplement their income more with public transfers and tax allowances. This applies especially where the tax-benefit system is strongly redistributive, as is the case in Northern and Northwestern Europe (Avram et al., 2014). An additional argument might be that women in low-income unions earn more similarly to their male partners than women in high-income unions. This is the dominant argument in the United States (Qian, 2017; Winslow-Bowe, 2006), though it is unclear whether this pattern holds true in Europe, where generous public transfers and expensive childcare encourage women in the lower socioeconomic strata to specialize in unpaid work (Evertsson et al., 2009). Because of the greater contribution of public transfers (and possibly women’s earnings) in lower-income unions, earnings of the male partner make up a smaller portion of the total household income. These arguments suggest that women in lower-income unions are relatively more independent of their partner, which could explain why living standards converge following union dissolution.

All in all, this leads to the following expectations (Table 1). I expect women from higher-income unions to have a compensatory advantage regarding personal earnings and tax payments, neither an advantage nor disadvantage regarding household composition and new partner earnings, and a compensatory disadvantage regarding transfer income. Furthermore, I expect women from higher-income unions to depend relatively more on the earnings of their initial partner, and this will outweigh all other effects.

**Table 1:** Theoretical expectations of changes in income components

Mechanism	Income component	Direction
Compensation	personal earnings	+
Compensation	tax payments	+
Compensation	household composition	o
Compensation	new partner earnings	o
Compensation	transfer income	−
Partner independence	ex-partner earnings	− –

+ more favorable to women from higher-income unions, o no socioeconomic gradient, – more favorable to women from lower-income unions

## The Dutch Context

The consequences of union dissolution were examined using the Netherlands as a case study. Approximately three quarters of the Dutch working-age population co-reside with a partner (Statistics Netherlands, 2022). Of these, 13% live in non-contractual cohabitation, 13% in contractual cohabitation, 4% in a registered partnership, and 70% in marriage (Poortman & Mills, 2012; Statistics Netherlands, 2022). In the first year of co-residence, only 18% are registered partners or married spouses. Fiscal differences between union types are minimal (Christl et al., 2021). Legal differences between cohabitation, on the one hand, and registered partnership and marriage, on the other, concern mainly pensions and inheritance. Registered partnership and marriage are legally equivalent (Perelli-Harris & Gassen, 2012).

The net divorce rate in the Netherlands is 45 divorces per 100 marriages, similar to that in Norway (44) and the United Kingdom (47) and around the European average (Eurostat, 2021). The dissolution of unmarried cohabitation is also around the European average (Kaplan & Stier, 2017). Dissolution is more common for unions that are formed at a young age, with partners who are born abroad, in which the woman out-earns the man, without children, and with a lower household income. For example, a standard deviation decrease in household income increases the odds of dissolution by almost a third (Kalmijn et al., 2007).

The financial consequences of union dissolution in the Netherlands have been studied by Manting and Bouman (2006). They found that men are on average unaffected, whereas women witness drops in living standards of 17% in the first year after dissolution and continue to be disadvantaged in the five years after. These drops are slightly less severe than those in other European countries (Uunk, 2004). If children are involved, legal custody is by default exercised jointly, though in practice 71% of children reside with their mother, 25% are in shared residence, and 5% reside with their father (Poortman & Van Gaalen, 2017). Shared residence is more common among ex-partners with higher incomes (Bakker & Mulder, 2013).

Incomes in the Netherlands are heavily influenced by the welfare state. Public transfers contribute 80% of gross household income in the bottom quintile, 60% in the second quintile, 35% in the third quintile, 15% in the fourth quintile, and 10% in the top quintile (Statistics Netherlands, 2012). Transfers take place primarily at the couple level. For instance, social assistance, healthcare benefits, housing benefits, child assistance, and childcare subsidies are means-tested at the couple level, and child benefits are a universal transfer at the child level. Transfers from unemployment, sickness, and disability insurances are granted at the individual level, but their use is limited, their duration is maximized at two years, and they are included in couple means tests. In addition to transfers, redistribution is achieved by taxation. The Netherlands has a relatively progressive tax scheme (Causa & Nørlem Hermansen, 2019). Taxation is fully individual, with the exception of small allowances on property taxes at the couple level. There is no distinction by marital status, as fiscal unity is imposed on nearly all couples (Christl et al., 2021).^1^

Private transfers play a minor role in the Netherlands. Partner alimony and child support contribute less than 1% to gross household income (Statistics Netherlands, 2012). Of these, partner alimony is the largest component and incorporated in the tax records, whereas child support is a small component and not observed. Indeed, Dutch child support hardly improves the living standards of separated women (Hogendoorn et al., 2020). Monetary transfers from older parents to their working-age children are infrequent (Dimova & Wolff, 2011). Co-residence with extended family or friends, and the associated access to income, is rare compared to other countries and episodes are typically short lived (Das et al., 2017).

## Method

### Data

I used longitudinal data from the Dutch administrative registers. These data cover all residents in the Netherlands and combined information from the population register, education registers, social insurance bank, and tax records. I followed coresidential unions formed between 2003 and 2005 for ten years, the period during which household incomes were available without a break in definition.

The study population was selected from the union file. This unique file identifies coresidential unions by joint residential moves, marriage, registered partnership, fiscal partnership, parent–child relationships, joint home ownership, joint participation in a pension fund, or the request of couple-level benefits. This results in high coverage, where the only unions that are not covered are those that have never moved, have not registered their relationship, do not have children, do not own a home, and have not requested any joint income provisions.^2^

I selected all heterosexual unions formed by women between age 21 and 55 (*N* = 338,664).^3^ The lower bound represents the age until which individuals can legally claim maintenance from their parents, the upper bound the age from which early retirement schemes are available. I restricted the sample to women outside of full-time education, because incomes are little indicative of students’ economic standing (*N* = 293,641). I only included women whose unions survived at least two full years, because the analysis required at least two observations before dissolution (*N* = 271,215). From these, I selected women who experienced union dissolution within ten years since union formation (*N* = 61,961). These women were followed from union formation, through union dissolution, until the end of the ten-year observation period or until censoring (by emigration, death, widowhood, or reaching age 55). The dissolution year itself was excluded, because many women accidentally listed their ex-partner on the tax returns of that calendar year. This resulted in a population of 590,766 person-years nested in 60,989 persons. After list-wise deletion of missing values, the population comprised 547,937 person-years nested in 60,394 persons.^4^

### Measures

*Union dissolution* was measured as the termination of a coresidential union, following a separation from the household by one or both partners. Living-apart-together relationships were not considered and living-together-apart relationships could not be distinguished. In case a couple interrupted their union for less than a year, this interruption was disregarded, since interruptions within a calendar year could not be distinguished in the tax records. *Time since union dissolution* was measured as the number of calendar years since union dissolution. *Household disposable income* was measured as the annual sum of labor earnings, business income, and investment income of all household members, after taxes and transfers, in euros. All incomes were inflated to their 2015 values (the last year in the data). Household income was adjusted for household composition and economies of scale using an empirically grounded equivalence scale for the Netherlands. This equivalence scale assigned a weight of 1 to each adult and a weight of 0.8 to each child under eighteen, after which each person was awarded the total household income divided by the square root of the weighted household size (Siermann et al., 2004).^5^*Pre-dissolution income group* measured the relative income position before dissolution. Within each union cohort and at each union duration, I selected all women who had not yet separated and all women who would not separate within ten years, and divided their household disposable incomes into quintiles. Each separating woman was then assigned the quintile corresponding to her income in the second year before dissolution.

Household disposable income was disaggregated into the following components. *First partner earnings* comprised the gross labor earnings and business income of the male partner with whom the woman was initially in a union. *Personal earnings* comprised the gross labor earnings and business income of the focal woman. *New partner earnings* comprised the gross labor earnings and business income of new partners, if any. *Other income* comprised the gross earnings and business income of other household members as well as (usually minor) dividends and capital gains and (negative) interest on mortgages. *Transfer income* comprised social security income and (usually minor) partner alimony of the household. *Taxes and contributions* comprised all income taxes and social security contributions of the household.

The analysis also included several other variables. *Age* was measured in years. *Nativity* was a binary indicator of Dutch-born versus foreign-born. *Working hours* was measured in full-time equivalents (fte): the number of hours in paid work as a proportion of full-time paid work. *Marital status* was a binary indicator of cohabitation versus registered partnership or marriage. *Adults* were measured as the number of resident persons aged 18 and over. *Children* were measured as the number of resident persons aged less than 18. *Union duration* was measured as the number of calendar years since union formation. *Unemployment rate* was measured as the annual rate of unemployment in the population aged 21–55.

Table 2 describes the population two years before union dissolution. Differences between income groups were most pronounced concerning nativity, working hours, children, and income sources. Women in lower-income groups were more often born abroad, worked fewer hours, and had more children than women in higher-income groups. They and especially their partners also earned less, received more transfers, and paid fewer taxes. These descriptives give a first indication that women in lower-income unions depended relatively little on their partner, because the welfare state provided a relatively large share of their household income.

**Table 2:** Descriptive statistics two years before union dissolution

	All	By pre-dissolution income group				
		Q1	Q2	Q3	Q4	Q5
*Individual characteristics*						
Age	33.87	34.04	33.57	33.17	33.71	35.35
	(7.50)	(7.38)	(7.49)	(7.62)	(7.63)	(7.14)
Foreign-born	0.22	0.36	0.22	0.17	0.13	0.14
Full-time equivalent	0.63	0.34	0.61	0.74	0.82	0.86
	(0.37)	(0.36)	(0.33)	(0.30)	(0.26)	(0.24)
*Union characteristics*						
Married	0.40	0.46	0.43	0.38	0.34	0.36
Adults	2.07	2.00	2.08	2.12	2.12	2.08
	(0.49)	(0.51)	(0.50)	(0.50)	(0.49)	(0.42)
Children	0.91	1.42	1.05	0.71	0.51	0.44
	(1.02)	(1.12)	(0.97)	(0.89)	(0.80)	(0.78)
Union duration	3.27	3.05	3.19	3.25	3.43	3.66
	(2.55)	(2.52)	(2.54)	(2.56)	(2.54)	(2.54)
*Income components*						
First partner earnings	42,447	18,068	34,143	43,421	54,658	88,200
	(38,938)	(20,862)	(21,502)	(26,614)	(31,555)	(61,903)
Personal earnings	26,954	9289	20,347	28,847	38,141	56,238
	(24,594)	(12,706)	(14,838)	(16,235)	(18,569)	(36,038)
New partner earnings	0	0	0	0	0	0
	(0)	(0)	(0)	(0)	(0)	(0)
Other income	− 3615	− 2288	− 3395	− 3905	− 4279	− 5338
	(15,963)	(9014)	(9585)	(12,661)	(21,408)	(27,545)
Transfer income	6380	10,202	6509	4950	3981	3844
	(10,873)	(10,967)	(10,035)	(10,060)	(9,899)	(12,274)
Taxes and contributions	− 31,008	− 11,511	− 22,669	− 31,290	− 42,217	− 69,421
	(25,445)	(8,539)	(9,714)	(12,506)	(16,820)	(38,319)
Household disposable income	25,774	13,651	20,943	26,413	32,591	48,524
	(15,498)	(8,640)	(5,154)	(9,715)	(9,680)	(21,065)
*N* persons	60,394	15,957	13,727	12,393	10,349	7,968

### Matching

The analysis examined differentials in the dissolution penalty. To estimate the dissolution penalty, I employed a difference-in-differences design. I compared women who separated to similar women who had a partner. For example, if separating women lost 20% of their income upon union dissolution and partnered women gained 5% in the same year, the dissolution penalty would amount to 25% points.

The difference-in-differences design hinged on the comparability of separating and partnered women. To ensure comparability, I used a matching approach. Each woman who separated was matched to a similar woman who was (still) partnered. The matching was exact on the variables pre-dissolution income group, union duration, nativity, marital status, children, and adults. The matching was probabilistic on the variables age, working hours, partner earnings, personal earnings, other income, transfer income, and taxes and contributions. That is, within each exact matching stratum, I computed the Mahalanobis distance between all separating women and their potential matches, discarded potential match pairs for whom the Mahalanobis distance exceeded 2 units or for whom the difference in household disposable income exceeded 1 standard deviation, and selected the nearest neighbor as the match. This form of near-exact matching ensured both univariate balance (i.e., group means) and multivariate balance (i.e., individual scores) of separating and partnered women, reducing model dependence and residual variance (Iacus et al., 2012).

Previous work has shown that some women anticipate the financial consequences of union dissolution by increasing their employment in the year before dissolution (Poortman, 2005; Thielemans & Mortelmans, 2019, 2022). To allow for anticipation effects, separating women were matched using their characteristics in the second year before dissolution. For partnered women, I used their characteristics at the corresponding union duration. (Separating and partnered women were matched at the same union duration, so their clocks were synchronized.)

Following this procedure, 96% of all separating women found a suitable match. Some partnered women appeared as matches multiple times because I allowed for replacement; standard errors were adjusted accordingly. The resulting dataset contained a treatment group of 527,648 person-years nested in 57,960 separating persons and a control group of 527,648 person-years nested in 44,172 partnered persons. The treatment and control group were well balanced on all covariates (see Table 3 of the Appendix). To enable others to replicate the analysis, I uploaded the replication files to the Open Science Framework (https://osf.io/vnufm/).

**Table 3:** Balance on matching variables between treatment and control group, two years before union dissolution

	Q1		Q2		Q3		Q4		Q5	
	Treat	Control	Treat	Control	Treat	Control	Treat	Control	Treat	Control
*Individual characteristics*										
Age	33.94	33.69	33.43	33.19	33.00	32.80	33.46	33.25	34.88	34.66
	(7.32)	(6.79)	(7.39)	(6.89)	(7.52)	(7.08)	(7.49)	(7.02)	(6.89)	(6.36)
Foreign-born	0.36	0.36	0.21	0.21	0.16	0.16	0.12	0.12	0.12	0.12
	(0.48)	(0.48)	(0.41)	(0.41)	(0.36)	(0.36)	(0.32)	(0.32)	(0.32)	(0.32)
Full-time equivalent	0.34	0.33	0.61	0.61	0.75	0.75	0.83	0.83	0.88	0.88
	(0.36)	(0.36)	(0.33)	(0.33)	(0.29)	(0.29)	(0.24)	(0.24)	(0.22)	(0.21)
*Union characteristics*										
Married	0.47	0.47	0.43	0.43	0.38	0.38	0.34	0.34	0.35	0.35
	(0.50)	(0.50)	(0.50)	(0.50)	(0.48)	(0.48)	(0.47)	(0.47)	(0.48)	(0.48)
Adults	2.00	2.00	2.07	2.06	2.10	2.10	2.09	2.09	2.05	2.05
	(0.48)	(0.48)	(0.45)	(0.44)	(0.45)	(0.45)	(0.41)	(0.41)	(0.29)	(0.30)
Children	1.42	1.42	1.05	1.05	0.70	0.70	0.49	0.49	0.40	0.40
	(1.12)	(1.11)	(0.96)	(0.95)	(0.88)	(0.87)	(0.78)	(0.79)	(0.74)	(0.74)
Union duration	3.04	3.04	3.19	3.19	3.25	3.25	3.42	3.42	3.63	3.63
	(2.52)	(2.52)	(2.54)	(2.54)	(2.56)	(2.56)	(2.54)	(2.54)	(2.55)	(2.55)
*Income components*										
First partner earnings	18,349	18,994	34,664	35,631	43,527	44,360	54,563	54,954	84,007	83,757
	(19,918)	(19,800)	(20,591)	(19,595)	(21,118)	(19,942)	(24,219)	(22,602)	(41,511)	(39,785)
Personal earnings	9259	9128	20,422	20,434	29,069	29,142	38,336	38,327	55,242	55,333
	(12,142)	(11,914)	(14,464)	(14,041)	(15,584)	(14,891)	(17,217)	(16,535)	(27,950)	(27,145)
Other income	− 2088	− 1947	− 3656	− 3741	− 4427	− 4513	− 5327	− 5367	− 7908	− 7828
	(5644)	(4897)	(7,889)	(7287)	(10,203)	(9616)	(11,532)	(10,887)	(13,568)	(13,026)
Transfer income	10,129	9913	6161	5813	4364	4032	3235	2954	2409	2138
	(10,800)	(10,741)	(9372)	(9140)	(8368)	(8112)	(7879)	(7614)	(7143)	(6905)
Taxes and contributions	− 11,403	− 11,529	− 22,576	− 23,010	− 31,004	− 31,459	− 41,629	− 41,775	− 65,497	− 65,476
	(8005)	(7692)	(9075)	(8224)	(10,392)	(9509)	(12,485)	(11,595)	(25,957)	(24,741)
Household disposable income	13,968	14,164	21,028	21,112	26,220	26,241	32,162	32,117	45,850	45,623
	(4721)	(4249)	(3456)	(2900)	(3654)	(3025)	(4231)	(3661)	(11,627)	(11,061)
*N* persons	15,546	15,546	13,334	13,334	11,967	11,967	9,896	9,896	7217	7217

Exact matching strata formed by pre-dissolution income group, union duration, nativity, marital status, children (top-coded at three), and adults (top-coded at three). Within these strata, 1-nearest-neighbor matching using the Mahalanobis distance based on age, working hours, first partner earnings, personal earnings, other income, transfer income, and taxes and contributions. Matches outside the caliper of 2 distance units or with a difference in household disposable income exceeding 1 standard deviation were discarded

### Estimation

The analysis aimed to decompose the dissolution penalty into changes in household composition and changes in income sources. To do so, I had to make several decisions. One decision regarded the way in which to untangle the dissolution penalty. Recall that household disposable income ${Y}_{it}$ is the sum of all income sources ${S}_{it}^{k}$ divided by an equivalence factor ${f}_{it}$:

$${Y}_{it}=\frac{{S}_{it}^{1}+{S}_{it}^{2}+{S}_{it}^{3}+\dots }{{f}_{it}}$$

This shows that union dissolution affects household disposable income via changes in household composition, captured by the denominator ${f}_{it}$, and via changes in income sources, captured by the numerators ${S}_{it}^{k}$. Because it is not possible to untangle changes in the denominator and numerators within one framework, I divided the analysis in two parts. The first part considered changes in household composition, without yet considering changes in income sources. The second part then considered changes in income sources, conditional on changes in household composition.

Another decision regarded the definition of relative income. I applied a proportional definition (Tach & Eads, 2015):

$${p}_{it}^{Y}=\frac{{Y}_{it}}{{\mathrm{abs}(Y}_{i,-2})}$$

Here, ${p}_{it}^{Y}$ was the proportion of household disposable income $Y$ in year *t* compared to the second year before dissolution $t=-2$. Incomes were anchored in the second year before dissolution, rather than the actual year of dissolution, to avoid anticipation effects in the baseline. The absolute value function ensured that gains and losses appeared as positive and negative changes, respectively. While relative income changes could also be modeled using a standard logarithmic definition, I preferred the proportional definition, because proportions are additively decomposable:

$$\frac{{Y}_{it}}{\mathrm{abs}\left({Y}_{i,-2}\right)}=\frac{{S}_{it}^{1} {/ f}_{it} }{\mathrm{abs}\left({Y}_{i,-2}\right)}+\frac{{S}_{it}^{2}{ / f}_{it}}{\mathrm{abs}\left({Y}_{i,-2}\right)}+\frac{{S}_{it}^{3}{ / f}_{it}}{\mathrm{abs}\left({Y}_{i,-2}\right)}+\dots {=p}_{it}^{Y}={p}_{it}^{{S}^{1}}+{p}_{it}^{{S}^{2}}+{p}_{it}^{{S}^{3}}+\dots$$

A last decision regarded the estimator. I used fixed-effects regressions to estimate the dissolution penalty. Fixed-effects regressions consider within-individual change over time, that is, how a transition into union dissolution is accompanied by a change in proportional income. A benefit is that time-invariant unobserved heterogeneity is automatically controlled for. A downside is that the estimates are sensitive to outliers: income losses might be underestimated if a few women experience extreme income gains following dissolution (Tach & Eads, 2015). To deal with this, I identified outliers using robust regression techniques for panel data. I temporarily subtracted the within-individual medians of proportional household disposable income and its explanatory variables, fitted a regression model to the de-medianed scores using the MS-estimator, and obtained the standardized residuals (Verardi & Wagner, 2011). The 1% most extreme residuals were eliminated, and models were run as usual.

Having made these decisions, the analysis proceeded as follows. First, I established the dissolution penalty. To do so, I estimated the following model:

$$\begin{array}{c}{p}_{it}^{Y}={\alpha }_{i}+{\varvec{\beta}} {{\varvec{T}}}_{{\varvec{i}}{\varvec{t}}}{{\varvec{Q}}}_{{\varvec{i}}}+{\varvec{\gamma}} {D}_{i}{{\varvec{T}}}_{{\varvec{i}}{\varvec{t}}}{{\varvec{Q}}}_{{\varvec{i}}}+{\varvec{\delta}} {U}_{it}{{\varvec{Q}}}_{{\varvec{i}}}+{\varepsilon }_{it}\end{array}$$

Here,${p}_{it}^{Y}$ was the proportion of household disposable income, ${\alpha }_{i}$ an individual intercept, ${{\varvec{T}}}_{{\varvec{i}}{\varvec{t}}}$ time since union dissolution dummies, ${{\varvec{Q}}}_{{\varvec{i}}}$ pre-dissolution income quintile dummies, ${D}_{i}$ a dummy indicating treatment or control group, ${U}_{it}$ the annual unemployment rate, and ${\varepsilon }_{it}$ an idiosyncratic error term.^6^ Women in the control group were assigned the dissolution year of their match. The coefficients ${\varvec{\gamma}}$ estimated the dissolution penalty. As outlined in the theory section, the penalty was expected to be larger for women from higher-income unions.

Next, I established the role of changes in household composition in the dissolution penalty, without yet considering changes in income sources. This situation could not be observed in the data since, in real life, both household composition and income sources change upon dissolution. To solve this, I assigned each woman in the treatment group the total (unequivalized) income of her match in the control group, and divided this income by her own equivalence factor. I then used this hypothetical situation to estimate the following model:

$$\begin{array}{c}\stackrel{\sim }{{p}_{it}^{Y}}={\alpha }_{i}+{\varvec{\beta}} {{\varvec{T}}}_{{\varvec{i}}{\varvec{t}}}{{\varvec{Q}}}_{{\varvec{i}}}+{\varvec{\gamma}} {D}_{i}{{\varvec{T}}}_{{\varvec{i}}{\varvec{t}}}{{\varvec{Q}}}_{{\varvec{i}}}+{\varvec{\delta}} {U}_{it}{{\varvec{Q}}}_{{\varvec{i}}}+{\varepsilon }_{it}\end{array}$$

Here,$\stackrel{\sim }{{p}_{it}^{Y}}$ was the proportion of household disposable income based on the observed household composition, observed income sources before dissolution, and hypothetical income sources after dissolution. The coefficients ${\varvec{\gamma}}$ estimated the dissolution penalty if only the household composition had changed. As outlined in the theory section, no difference was expected between women from low-income and women from high-income unions.

Finally, I established the role of changes in income sources in the dissolution penalty, given any changes in household composition. To do so, I estimated the following model:

$$\begin{array}{c}{p}_{it}^{{S}^{k}}={\alpha }_{i}+{\varvec{\beta}} {{\varvec{T}}}_{{\varvec{i}}{\varvec{t}}}{{\varvec{Q}}}_{{\varvec{i}}}+{\varvec{\gamma}} {D}_{i}{{\varvec{T}}}_{{\varvec{i}}{\varvec{t}}}{{\varvec{Q}}}_{{\varvec{i}}}+{\varvec{\delta}} {U}_{it}{{\varvec{Q}}}_{{\varvec{i}}}+{\varepsilon }_{it}\end{array}$$

Here, ${p}_{it}^{{S}^{k}}$ was each (equivalized) income component of household disposable income ${p}_{it}^{Y}$. The coefficients ${\varvec{\gamma}}$ estimated the dissolution effect on each income component. As outlined in the theory section, the effects on personal earnings and taxes were expected to be more positive for women from higher-income unions, the effect on new partner earnings to be similar across unions, and the effects on transfers and first partner earnings to be more negative for women from higher-income unions, with the effect on first partner earnings outweighing all other effects.

The results yielded a decomposition of the dissolution penalty. For each component, they showed what separating women lost compared to a situation in which they would have remained partnered, holding constant the other components. This gave insight into the relative importance of each component for the financial consequences of union dissolution.

## Results

### Dissolution Penalty

**Fig. 1:**
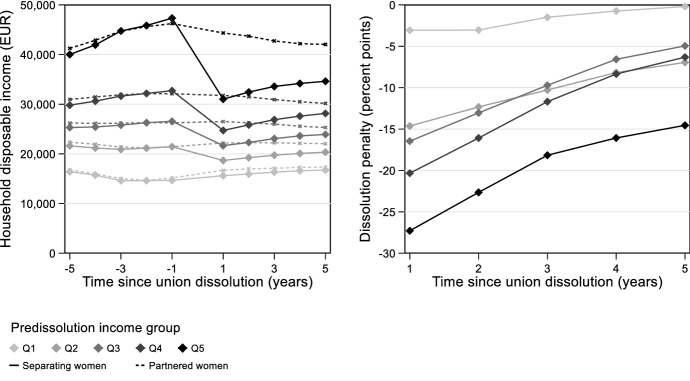
Observed changes in household disposable income for separating women and matched partnered women **(a)** and fixed-effects estimates of the dissolution penalty **(b)**. *Notes:* The dissolution penalty estimates are based on a matched sample and are controlled for individual fixed effects, time since union dissolution dummies, the national unemployment rate, and all their interactions with pre-dissolution income group. Complete model estimates are available in Table 4 of the Appendix

The first part of the analysis established the dissolution penalty. Figure 1a shows the observed development of household disposable income for separating women in the years before and after dissolution, comparing it to the matched group of partnered women. A clear gradient emerged, in which women in higher-income unions had higher incomes and diverged from women in lower-income unions as their unions progressed. This divergence came to an end upon dissolution. Women from higher-income unions lost considerably more than women in lower-income unions, resulting in income convergence.

These observations were confirmed by the fixed-effects model. Figure 1b shows the estimated dissolution penalty. The penalty was considerably larger for women from higher-income unions. One year after dissolution, women in the bottom quintile had lost 3 percentage points of income compared to their partnered matches. This number amounted to 15 points in the second quintile, 16 points in the third quintile, 20 points in the fourth quintile, and 27 points in the top quintile. These numbers confirm that women’s living standards converged upon dissolution.

Moreover, the convergence persisted as time passed. Women across all income groups recovered in the years after dissolution. And although women in higher-income groups recovered slightly faster, the convergence brought about by dissolution could still be noticed many years later.

The figure also reveals some results that are not directly related to the research question, yet that are worth mentioning. Firstly, the pre-dissolution income trends of separating women and their partnered matches were reasonably similar. This provided support for the common trends assumption of the difference-in-differences design. Besides, the incomes of the higher income groups took a small hike in the year before dissolution. This could represent an anticipation effect, though it could also relate to unexpected financial change that triggered dissolution (Böheim & Ermisch, 2001; Folke & Rickne, 2020). Lastly, income drops among separating women were followed by (smaller) income drops among their partnered matches. This could stem from the characteristics of partnered women but also from the fact that some of them (12%) separated a few years after their match did.

### Household Composition

**Fig. 2:**
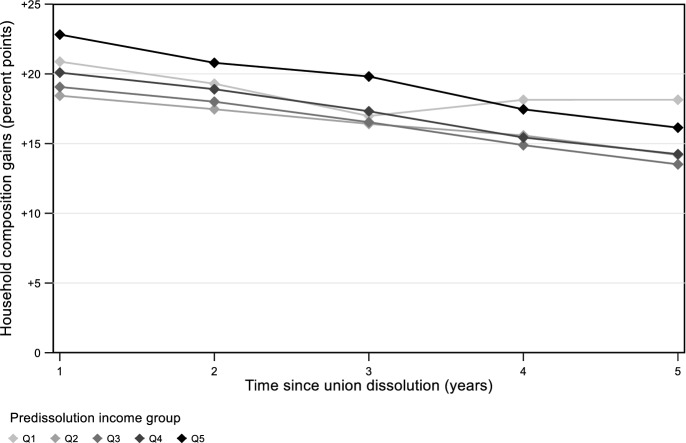
The financial gains of changes in household composition following union dissolution. *Notes:* Complete model estimates are available in Table 4 of the Appendix

The next part of the analysis established the role of compensation for the convergence in women’s living standards. One factor in the compensation mechanism is household composition. Figure 2 shows the financial gains of changes in household composition, without yet considering changes in income sources. The figure reveals that changes in household composition benefited women across all pre-dissolution income groups. One year after dissolution, women in the bottom quintile would have gained 21 percentage points compared to their partnered matches if they had reduced their household size but not their income sources. This number amounted to 18 points in the second quintile, 19 points in the third quintile, 20 points in the fourth quintile, and 23 points in the top quintile.

The financial gains of changes in household composition slowly faded as time passed. This was because some separated women found a new partner, expanding their households, and because some partnered women watched their children leave the household, contracting their households. In any case, the gains remained similar across pre-dissolution income groups, so household composition could not explain why women’s living standards converged following union dissolution.

### Income Sources

**Fig. 3:**
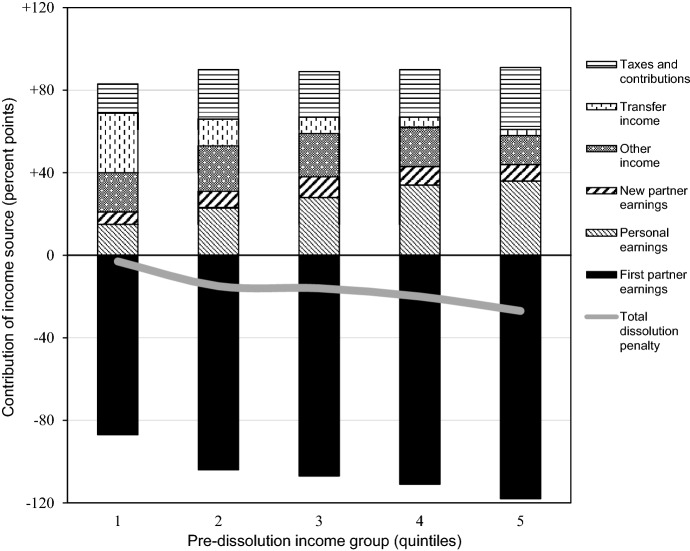
Fixed-effects decomposition of the dissolution penalty. *Notes:* Decomposition in the first year after dissolution, conditional on changes in household composition. Estimates may exceed ± 100 because they are pre-tax and pre-transfer; taxes and transfers are included as separate components. Complete model estimates are available in Table 4 of the Appendix

Another factor in the compensation mechanism is personal earnings. Figure 3 shows a decomposition of the dissolution penalty in the first year after union dissolution, given changes in household composition, with the part above the zero-line showing compensation via several income sources. The figure reveals that women compensated the loss of partner earnings with additional personal earnings, and this applied especially to women in higher-income unions. For instance, women in the bottom quintile increased their earnings by 15 percentage points compared to their partnered matches. This number amounted to 23 points in the second quintile, 28 points in the third quintile, 34 points in the fourth quintile, and 36 points in the top quintile. These numbers confirmed the expectation that women from high-income unions were better equipped to increase their personal earnings than women from low-income unions. Nonetheless, the pattern of earnings increases ran counter to the convergence in living standards, so earnings increases could not explain why women’s living standards converged following dissolution.

Women could also compensate losses by bringing in earnings from a new partner. Figure 3 reveals that some women benefited from this. New partner earnings increased by 6 percentage points in the bottom quintile, 8 points in the second quintile, 10 points in the third quintile, 9 points in the fourth quintile, and 8 points in the top quintile. However, the increases were small and did not differ much by pre-dissolution income group, meaning that repartnering could not explain why women’s living standards converged following dissolution.

Women could further compensate losses via “other” income sources. I did not formulate theoretical expectations here, but Fig. 3 reveals noticeable increases. Other income increased by 19 percentage points in the bottom quintile, 22 points in the second quintile, 21 points in the third quintile, 19 points in the fourth quintile, and 14 points in the top quintile. Although the data did not allow for detailed disaggregation, these figures probably reflected lower interest payments on mortgages. Following union dissolution, any mortgage passed to one of the ex-partners (most often the male partner, Kooiman, 2021), which lowered the interest paid by separated women. Even so, the benefits did not show a clear pattern across pre-dissolution income groups, implying that other income could not explain why women’s living standards converged following dissolution.

Could certain groups of women have a compensatory advantage via the tax-benefit system? Fig. 3 reveals that increases in transfers mainly benefited women from low-income unions. The amount of transfer income increased by 29 percentage points among women in the bottom quintile, 13 points in the second quintile, 8 points in the third quintile, 5 points in the fourth quintile, and 3 points in the upper quintiles. This is in line with the means-tested character of most transfers, whereby women with little income pass the threshold to qualify for welfare. Decreases in tax payments and social security contributions, on the other hand, were somewhat more beneficial to women from high-income unions. The portion of income spent on taxes decreased by 14 percentage points among women in the bottom quintile, 24 points in the second quintile, 22 points in the third quintile, 23 points in the fourth quintile, and 30 points in the top quintile. This is in line with the progressivity of the tax scheme, whereby the loss of a high-earning partner entails the end of his high tax payments. In other words, the tax-benefit system provided substantial compensation to women across pre-dissolution income groups, with a small overall advantage for women from lower-income unions. This means that taxes and transfers could not explain why women’s living standards converged following union dissolution.

Hence, I turned to the partner independence mechanism. As shown in Table 2, prior to dissolution, low-income unions supplemented their earnings with relatively much transfer income. Figure 3 shows what this meant for women who separated, as the part below the zero-line shows the relative losses of partner earnings upon dissolution. It turns out that women from higher-income unions lost a considerably larger share of household income by splitting from their partner. The contribution of partner earnings dropped by 87 percentage points among women in the bottom quintile, 104 points in the second quintile, 107 points in the third quintile, 111 points in the fourth quintile, and 118 points in the top quintile. This implies that the relative dependence on partner earnings was much greater for women in high-income unions than for women from low-income unions. Put differently, women from lower-income unions were relatively independent of their partner, which protected them against the financial consequences of union dissolution.

All in all, the results demonstrated the following with respect to compensation and partner independence. The compensation mechanism could not explain why women’s living standards converged following union dissolution. Women from all pre-dissolution income groups compensated losses with changes in household composition, increases in new partner earnings, and increases in other income; women from low-income unions further compensated with transfer income; and women in high-income unions further compensated with personal earnings and reductions in tax payments. Instead, partner independence was the dominant mechanism. Prior to dissolution, women in low-income unions depended relatively less on their partner’s contribution to the household than women in high-income unions, because their households received more transfer income. Consequently, the loss of partner earnings affected their standard of living relatively little.

**Table 4:** Fixed-effects regressions of changes in income components following union dissolution

	Household disp. income	Household composition	First partner earnings	Personal earnings	New partner earnings	Other income	Transfer income	Taxes and contribution
1st year before dissolution								
× treatment × Q1	− 0.01*	0.00	− 0.04***	0.02**	− 0.01*	0.00	0.00	0.01***
× treatment × Q2	0.03***	0.03***	0.01	0.03***	− 0.01***	− 0.01**	0.01***	− 0.01*
× treatment × Q3	0.03***	0.04***	0.03***	0.04***	− 0.01***	− 0.02***	0.01***	− 0.02***
× treatment × Q4	0.03***	0.04***	0.04***	0.05***	− 0.01***	− 0.02***	0.01**	− 0.03***
× treatment × Q5	0.04***	0.04***	0.05***	0.04***	− 0.01***	− 0.01**	0.00	− 0.04***
1st year after dissolution								
× treatment × Q1	− 0.03***	0.21***	− 0.87***	0.15***	0.06***	0.19***	0.29***	0.14***
× treatment × Q2	− 0.15***	0.18***	− 1.04***	0.23***	0.08***	0.22***	0.13***	0.24***
× treatment × Q3	− 0.16***	0.19***	− 1.07***	0.28***	0.10***	0.21***	0.08***	0.22***
× treatment × Q4	− 0.20***	0.20***	− 1.11***	0.34***	0.09***	0.19***	0.05***	0.23***
× treatment × Q5	− 0.27***	0.23***	− 1.18***	0.36***	0.08***	0.14***	0.03***	0.30***
2nd year after dissolution								
× treatment × Q1	− 0.03***	0.19***	− 0.85***	0.14***	0.13***	0.15***	0.26***	0.14***
× treatment × Q2	− 0.12***	0.17***	− 1.03***	0.21***	0.19***	0.16***	0.12***	0.22***
× treatment × Q3	− 0.13***	0.18***	− 1.04***	0.27***	0.22***	0.16***	0.07***	0.18***
× treatment × Q4	− 0.16***	0.19***	− 1.09***	0.35***	0.23***	0.13***	0.04***	0.18***
× treatment × Q5	− 0.23***	0.21***	− 1.14***	0.35***	0.18***	0.12***	0.03***	0.25***
3rd year after dissolution								
× treatment × Q1	− 0.01*	0.17***	− 0.83***	0.11***	0.23***	0.11***	0.25***	0.12***
× treatment × Q2	− 0.10***	0.16***	− 1.00***	0.19***	0.30***	0.11***	0.11***	0.19***
× treatment × Q3	− 0.10***	0.17***	− 1.02***	0.27***	0.35***	0.11***	0.06***	0.14***
× treatment × Q4	− 0.12***	0.17***	− 1.07***	0.33***	0.37***	0.10***	0.03***	0.12***
× treatment × Q5	− 0.18***	0.20***	− 1.12***	0.33***	0.31***	0.08***	0.03***	0.20***
4th year after dissolution								
× treatment × Q1	− 0.01	0.18***	− 0.81***	0.09***	0.31***	0.08***	0.22***	0.10***
× treatment × Q2	− 0.08***	0.16***	− 0.97***	0.17***	0.37***	0.09***	0.10***	0.16***
× treatment × Q3	− 0.07***	0.15***	− 0.99***	0.24***	0.45***	0.08***	0.06***	0.10***
× treatment × Q4	− 0.08***	0.15***	− 1.05***	0.31***	0.47***	0.08***	0.02***	0.09***
× treatment × Q5	− 0.16***	0.17***	− 1.11***	0.30***	0.38***	0.07***	0.02***	0.17***
5th year after dissolution								
× treatment × Q1	0.00	0.18***	− 0.77***	0.06***	0.36***	0.05***	0.20***	0.09***
× treatment × Q2	− 0.07***	0.14***	− 0.95***	0.16***	0.43***	0.06***	0.09***	0.14***
× treatment × Q3	− 0.05***	0.14***	− 0.97***	0.22***	0.51***	0.06***	0.06***	0.08***
× treatment × Q4	− 0.06***	0.14***	− 1.04***	0.27***	0.55***	0.05***	0.03***	0.08***
× treatment × Q5	− 0.15***	0.16***	− 1.11***	0.28***	0.46***	0.05***	0.02***	0.17***
6th year after dissolution								
× treatment × Q1	0.00	0.16***	− 0.74***	0.04*	0.40***	0.03**	0.19***	0.09***
× treatment × Q2	− 0.06***	0.13***	− 0.93***	0.14***	0.46***	0.05***	0.10***	0.13***
× treatment × Q3	− 0.04***	0.13***	− 0.94***	0.20***	0.55***	0.04***	0.05***	0.06***
× treatment × Q4	− 0.05***	0.13***	− 1.02***	0.24***	0.60***	0.03***	0.03***	0.07***
× treatment × Q5	− 0.13***	0.16***	− 1.09***	0.27***	0.49***	0.04***	0.02***	0.14***
7th year after dissolution								
× treatment × Q1	0.01	0.15***	− 0.69***	0.03	0.39***	0.02	0.16***	0.10***
× treatment × Q2	− 0.05***	0.13***	− 0.90***	0.12***	0.48***	0.04***	0.09***	0.12***
× treatment × Q3	− 0.03***	0.12***	− 0.91***	0.17***	0.58***	0.01	0.06***	0.06***
× treatment × Q4	− 0.05***	0.12***	− 0.99***	0.20***	0.61***	0.02*	0.03***	0.08***
× treatment × Q5	− 0.13***	0.15***	− 1.09***	0.24***	0.52***	0.02*	0.02**	0.16***
8th year after dissolution								
× treatment × Q1	0.01	0.14***	− 0.63***	0.01	0.41***	− 0.02	0.16***	0.09***
× treatment × Q2	− 0.05***	0.13***	− 0.87***	0.10***	0.48***	0.02	0.10***	0.11***
× treatment × Q3	− 0.04***	0.11***	− 0.89***	0.13***	0.60***	0.01	0.07***	0.05***
× treatment × Q4	− 0.04***	0.11***	− 0.98***	0.19***	0.62***	0.02	0.03***	0.07***
× treatment × Q5	− 0.12***	0.16***	− 1.07***	0.21***	0.56***	0.01	0.02*	0.14***
Unemployment rate								
× Q1	− 0.05***	− 0.03***	− 0.06***	− 0.02***	− 0.01***	0.01***	0.02***	0.02***
× Q2	− 0.03***	− 0.01***	− 0.05***	− 0.01***	− 0.01***	0.01***	0.01***	0.02***
× Q3	− 0.02***	− 0.01***	− 0.04***	− 0.01***	− 0.01***	0.01***	0.01***	0.02***
× Q4	− 0.02***	− 0.01***	− 0.03***	− 0.02***	− 0.01***	0.01***	0.01***	0.02***
× Q5	− 0.03***	− 0.01***	− 0.03***	− 0.02***	− 0.01***	0.01***	0.01***	0.01***
Time × Q dummies	✓	✓	✓	✓	✓	✓	✓	✓
Individual FE	✓	✓	✓	✓	✓	✓	✓	✓
*N* unique persons	96,791	96,791	96,791	96,791	96,791	96,791	96,791	96,791
*N* persons	115,775	115,775	115,775	115,775	115,775	115,775	115,775	115,775
*N* person-years	1,044,672	1,044,672	1,044,672	1,044,672	1,044,672	1,044,672	1,044,672	1,044,672
*R*^2^ overall	0.98	0.91	0.85	0.94	0.69	0.47	0.92	0.87
*R*^2^ within	0.07	0.05	0.33	0.04	0.13	0.02	0.06	0.05
*F* group diff (1st year)	244***	13***	135***	107***	7***	22***	296***	55***
*F* group diff (2nd year)	132***	6***	102***	105***	26***	8***	244***	33***
*F* group diff (3rd year)	81***	5***	92***	88***	35***	4***	183***	26***
*F* group diff (4th year)	57***	4***	77***	80***	33***	1*	135***	25***
*F* group diff (5th year)	42***	6***	93***	61***	41***	1*	81***	20***
*F* group diff (6th year)	27***	3**	90***	34***	41***	1*	57***	14***
*F* group diff (7th year)	22***	3**	76***	27***	28***	1*	24***	11***
*F* group diff (8th year)	10***	2*	58***	15***	18***	2	17***	5***

Standard errors accounted for clustering at the individual level. *p < .05, **p < *.*01, ***p < *.*001

### Robustness Checks

Additional analyses confirmed the robustness of the results. A first analysis concerned the estimation. The previous sections showed the average effect of dissolution on proportional income, excluding the 1% most extreme outliers. This might not provide a good estimate of the “typical” dissolution penalty. Hence, I estimated several alternative models. I first re-ran the main model, excluding different percentages of outliers. This showed that excluding less than 0.5% would change the results notably, but that excluding more than 1% would make little difference. I then ran models that were less sensitive to outliers on the full sample, namely a fixed-effects regression of average log income and a quantile regression of median proportional income. The results were very similar to the main results (Table 5 of the Appendix).

**Table 5:** Fixed-effects regressions of change in household disposable income following union dissolution, using different model specifications

	FE p-hdi (full sample)	FE p-hdi (excl. 0.5%)	FE p-hdi (excl. 1%)	FE p-hdi (excl. 2%)	FE p-hdi (excl. 5%)	FE log-hdi (full sample)	QR p-hdi (full sample)
1st year before dissolution							
× treatment × Q1	0.02	− 0.02**	− 0.01*	0.00	0.00	− 0.02***	− 0.01***
× treatment × Q2	− 0.15	0.02***	0.03***	0.03***	0.03***	0.02***	0.01***
× treatment × Q3	0.03**	0.03***	0.03***	0.03***	0.03***	0.03***	0.01***
× treatment × Q4	0.04***	0.03***	0.03***	0.03***	0.03***	0.03***	0.01***
× treatment × Q5	0.04***	0.04***	0.04***	0.04***	0.03***	0.04***	0.01***
1st year after dissolution							
× treatment × Q1	0.18	− 0.02**	− 0.03***	− 0.04***	− 0.05***	− 0.04***	− 0.03***
× treatment × Q2	− 0.23**	− 0.14***	− 0.15***	− 0.15***	− 0.15***	− 0.17***	− 0.19***
× treatment × Q3	− 0.17***	− 0.16***	− 0.16***	− 0.16***	− 0.16***	− 0.19***	− 0.22***
× treatment × Q4	− 0.20***	− 0.20***	− 0.20***	− 0.20***	− 0.20***	− 0.23***	− 0.26***
× treatment × Q5	− 0.27***	− 0.28***	− 0.27***	− 0.27***	− 0.25***	− 0.31***	− 0.32***
2nd year after dissolution							
× treatment × Q1	0.18*	− 0.02**	− 0.03***	− 0.04***	− 0.04***	− 0.03***	− 0.02***
× treatment × Q2	− 0.20*	− 0.12***	− 0.12***	− 0.12***	− 0.12***	− 0.15***	− 0.17***
× treatment × Q3	− 0.14***	− 0.13***	− 0.13***	− 0.13***	− 0.13***	− 0.16***	− 0.19***
× treatment × Q4	− 0.16***	− 0.16***	− 0.16***	− 0.16***	− 0.16***	− 0.19***	− 0.22***
× treatment × Q5	− 0.22***	− 0.23***	− 0.23***	− 0.23***	− 0.21***	− 0.27***	− 0.27***
3rd year after dissolution							
× treatment × Q1	0.30*	0.00	− 0.01*	− 0.03***	− 0.04***	− 0.01*	− 0.01*
× treatment × Q2	− 0.19*	− 0.10***	− 0.10***	− 0.10***	− 0.10***	− 0.12***	− 0.14***
× treatment × Q3	− 0.11***	− 0.10***	− 0.10***	− 0.10***	− 0.10***	− 0.13***	− 0.15***
× treatment × Q4	− 0.11***	− 0.12***	− 0.12***	− 0.12***	− 0.11***	− 0.15***	− 0.15***
× treatment × Q5	− 0.19***	− 0.19***	− 0.18***	− 0.18***	− 0.17***	− 0.23***	− 0.23***
4th year after dissolution							
× treatment × Q1	0.32**	0.00	− 0.01	− 0.02***	− 0.03***	− 0.01	− 0.01
× treatment × Q2	− 0.19	− 0.08***	− 0.08***	− 0.09***	− 0.09***	− 0.10***	− 0.11***
× treatment × Q3	− 0.07***	− 0.06***	− 0.07***	− 0.07***	− 0.07***	− 0.09***	− 0.10***
× treatment × Q4	− 0.08***	− 0.08***	− 0.08***	− 0.09***	− 0.09***	− 0.12***	− 0.12***
× treatment × Q5	− 0.16***	− 0.16***	− 0.16***	− 0.16***	− 0.15***	− 0.21***	− 0.19***
5th year after dissolution							
× treatment × Q1	0.26*	0.02*	0.00	− 0.01*	− 0.03***	0.00	− 0.01
× treatment × Q2	− 0.19	− 0.07***	− 0.07***	− 0.07***	− 0.07***	− 0.08***	− 0.10***
× treatment × Q3	− 0.05***	− 0.05***	− 0.05***	− 0.05***	− 0.06***	− 0.08***	− 0.07***
× treatment × Q4	− 0.06***	− 0.06***	− 0.06***	− 0.07***	− 0.07***	− 0.10***	− 0.09***
× treatment × Q5	− 0.14***	− 0.15***	− 0.15***	− 0.14***	− 0.13***	− 0.19***	− 0.16***
6th year after dissolution							
× treatment × Q1	0.22	0.02	0.00	− 0.01	− 0.03***	0.00	− 0.02*
× treatment × Q2	− 0.13	− 0.06***	− 0.06***	− 0.07***	− 0.07***	− 0.08***	− 0.08***
× treatment × Q3	− 0.04***	− 0.04***	− 0.04***	− 0.04***	− 0.05***	− 0.07***	− 0.05***
× treatment × Q4	− 0.05***	− 0.05***	− 0.05***	− 0.06***	− 0.06***	− 0.08***	− 0.06***
× treatment × Q5	− 0.13***	− 0.13***	− 0.13***	− 0.13***	− 0.12***	− 0.17***	− 0.13***
7th year after dissolution							
× treatment × Q1	0.46**	0.02	0.01	− 0.01	− 0.03***	0.00	− 0.03*
× treatment × Q2	− 0.13	− 0.05***	− 0.05***	− 0.06***	− 0.06***	− 0.07***	− 0.06***
× treatment × Q3	− 0.03**	− 0.03***	− 0.03***	− 0.04***	− 0.04***	− 0.05***	− 0.02**
× treatment × Q4	− 0.03	− 0.05***	− 0.05***	− 0.06***	− 0.06***	− 0.09***	− 0.05***
× treatment × Q5	− 0.12***	− 0.13***	− 0.13***	− 0.13***	− 0.11***	− 0.17***	− 0.12***
8th year after dissolution							
× treatment × Q1	0.41*	0.03	0.01	− 0.01	− 0.02*	0.01	− 0.04*
× treatment × Q2	− 0.16	− 0.05***	− 0.05***	− 0.05***	− 0.07***	− 0.07***	− 0.05**
× treatment × Q3	− 0.03	− 0.03*	− 0.04***	− 0.04***	− 0.05***	− 0.06***	− 0.01
× treatment × Q4	− 0.04**	− 0.04**	− 0.04***	− 0.05***	− 0.05***	− 0.08***	− 0.02
× treatment × Q5	− 0.13***	− 0.11***	− 0.12***	− 0.13***	− 0.10***	− 0.16***	− 0.13***
Unemployment rate							
× Q1	− 0.11**	− 0.05***	− 0.05***	− 0.04***	− 0.03***	− 0.05***	− 0.01***
× Q2	0.00	− 0.03***	− 0.03***	− 0.03***	− 0.02***	− 0.03***	− 0.01***
× Q3	− 0.03***	− 0.02***	− 0.02***	− 0.02***	− 0.02***	− 0.03***	− 0.01***
× Q4	− 0.02***	− 0.02***	− 0.02***	− 0.02***	− 0.02***	− 0.03***	− 0.01***
× Q5	− 0.03***	− 0.03***	− 0.03***	− 0.02***	− 0.02***	− 0.03***	− 0.01***
Time × Q dummies	✓	✓	✓	✓	✓	✓	✓
Individual FE	✓	✓	✓	✓	✓	✓	
*N* unique persons	96,895	96,842	96,791	96,712	96,514	96,882	96,895
*N* persons	115,920	115,842	115,775	115,667	115,408	115,904	115,920
*N* person-years	1,055,296	1,049,983	1,044,672	1,034,078	1,002,287	1,050,746	1,055,296
*R*^2^ overall	0.83	0.97	0.98	0.98	0.93	0.66	
*R*^2^ within	0.00	0.07	0.07	0.08	0.10	0.08	
*F* group diff (1st year)	37***	203***	244***	294***	365***	340***	473***
*F* group diff (2nd year)	20***	109***	132***	160***	205***	200***	364***
*F* group diff (3rd year)	17***	75***	81***	91***	102***	136***	185***
*F* group diff (4th year)	21***	47***	57***	63***	64***	87***	86***
*F* group diff (5th year)	17***	41***	42***	45***	42***	64***	44***
*F* group diff (6th year)	9***	26***	27***	32***	27***	46***	14***
*F* group diff (7th year)	8***	19***	22***	25***	18***	32***	8***
*F* group diff (8th year)	5***	9***	10***	11***	11***	19***	5***

FE fixed effects, QR quantile regression, p-hdi proportional household disposable income, log-hdi natural logarithm of household disposable income. In parentheses the part of the dataset used for estimation. Standard errors accounted for clustering at the individual level. Coefficients of the log-income model were transformed as *e*^b^−1 to also show relative changes in income. *p < .05, **p < *.*01, ***p < *.*001

A second analysis concerned the equivalence scale. The previous sections relied on an empirically grounded scale used by Statistics Netherlands; other scales might give different results. Hence, I repeated the analysis using two commonly used equivalence scales. The OECD-modified scale assigns a weight of 1 to the first adult, 0.5 to each subsequent adult, and 0.3 to each child, whereas the square root scale takes the square root of unweighted household size. The results for these equivalence scales were very similar to the main results. The only difference was that the OECD-modified scale yielded a slightly smaller dissolution penalty across income groups, as this scale rewards reductions in household size more (Tables 6 and 7 of the Appendix).

**Table 6:** Fixed-effects regressions of changes in income components following union dissolution, using the OECD-modified equivalence scale

	Household disp. income	Household composition	First partner earnings	Personal earnings	New partner earnings	Other income	Transfer income	Taxes and contribution
1st year before dissolution								
× treatment × Q1	− 0.01**	0.00	− 0.04***	0.02**	− 0.01	− 0.01	0.01	0.01***
× treatment × Q2	0.03***	0.03***	0.01*	0.03***	− 0.01***	− 0.01**	0.01***	− 0.01*
× treatment × Q3	0.03***	0.04***	0.03***	0.04***	− 0.01***	− 0.02***	0.01***	− 0.02***
× treatment × Q4	0.03***	0.04***	0.04***	0.05***	− 0.01***	− 0.02***	0.01***	− 0.03***
× treatment × Q5	0.04***	0.04***	0.05***	0.04***	− 0.01***	− 0.01**	0.00	− 0.04***
1st year after dissolution								
× treatment × Q1	0.03***	0.23***	− 0.86***	0.18***	0.05***	0.19***	0.34***	0.12***
× treatment × Q2	− 0.09***	0.21***	− 1.04***	0.28***	0.08***	0.21***	0.15***	0.22***
× treatment × Q3	− 0.11***	0.22***	− 1.06***	0.34***	0.10***	0.21***	0.09***	0.19***
× treatment × Q4	− 0.16***	0.24***	− 1.11***	0.42***	0.09***	0.18***	0.06***	0.20***
× treatment × Q5	− 0.22***	0.27***	− 1.18***	0.45***	0.08***	0.14***	0.04***	0.25***
2nd year after dissolution								
× treatment × Q1	0.03***	0.21***	− 0.84***	0.16***	0.12***	0.15***	0.32***	0.12***
× treatment × Q2	− 0.08***	0.20***	− 1.02***	0.26***	0.18***	0.16***	0.14***	0.20***
× treatment × Q3	− 0.08***	0.21***	− 1.04***	0.34***	0.23***	0.16***	0.08***	0.15***
× treatment × Q4	− 0.12***	0.22***	− 1.09***	0.42***	0.23***	0.13***	0.04***	0.15***
× treatment × Q5	− 0.18***	0.24***	− 1.14***	0.43***	0.18***	0.11***	0.03***	0.20***
3rd year after dissolution								
× treatment × Q1	0.04***	0.18***	− 0.82***	0.12***	0.22***	0.11***	0.30***	0.10***
× treatment × Q2	− 0.06***	0.19***	− 0.99***	0.23***	0.29***	0.11***	0.13***	0.17***
× treatment × Q3	− 0.05***	0.19***	− 1.02***	0.32***	0.35***	0.11***	0.07***	0.11***
× treatment × Q4	− 0.08***	0.20***	− 1.07***	0.39***	0.38***	0.10***	0.04***	0.10***
× treatment × Q5	− 0.14***	0.23***	− 1.12***	0.41***	0.31***	0.07***	0.03***	0.16***
4th year after dissolution								
× treatment × Q1	0.04***	0.19***	− 0.79***	0.11***	0.29***	0.08***	0.27***	0.09***
× treatment × Q2	− 0.04***	0.17***	− 0.96***	0.20***	0.37***	0.09***	0.13***	0.14***
× treatment × Q3	− 0.03***	0.17***	− 0.99***	0.29***	0.45***	0.07***	0.07***	0.08***
× treatment × Q4	− 0.04***	0.17***	− 1.05***	0.36***	0.48***	0.07***	0.03***	0.06***
× treatment × Q5	− 0.12***	0.20***	− 1.10***	0.37***	0.38***	0.07***	0.03***	0.14***
5th year after dissolution								
× treatment × Q1	0.04***	0.19***	− 0.75***	0.07***	0.35***	0.05***	0.24***	0.07***
× treatment × Q2	− 0.03***	0.16***	− 0.93***	0.19***	0.43***	0.06***	0.11***	0.12***
× treatment × Q3	− 0.01**	0.15***	− 0.96***	0.26***	0.51***	0.06***	0.06***	0.05***
× treatment × Q4	− 0.03***	0.16***	− 1.04***	0.32***	0.55***	0.05***	0.04***	0.05***
× treatment × Q5	− 0.10***	0.18***	− 1.10***	0.34***	0.46***	0.05***	0.02***	0.13***
6th year after dissolution								
× treatment × Q1	0.04***	0.16***	− 0.72***	0.05*	0.38***	0.03*	0.23***	0.08***
× treatment × Q2	− 0.02**	0.15***	− 0.91***	0.17***	0.46***	0.05***	0.11***	0.10***
× treatment × Q3	0.00	0.14***	− 0.94***	0.24***	0.56***	0.03***	0.06***	0.04***
× treatment × Q4	− 0.02*	0.14***	− 1.01***	0.28***	0.60***	0.04***	0.04***	0.04***
× treatment × Q5	− 0.09***	0.18***	− 1.08***	0.32***	0.50***	0.04***	0.03***	0.11***
7th year after dissolution								
× treatment × Q1	0.04***	0.15***	− 0.68***	0.04*	0.38***	0.02	0.19***	0.09***
× treatment × Q2	− 0.02*	0.13***	− 0.87***	0.15***	0.47***	0.04***	0.10***	0.10***
× treatment × Q3	0.00	0.14***	− 0.90***	0.21***	0.58***	0.01	0.06***	0.03**
× treatment × Q4	− 0.02*	0.14***	− 0.98***	0.24***	0.61***	0.02*	0.04***	0.05***
× treatment × Q5	− 0.09***	0.17***	− 1.07***	0.29***	0.51***	0.02	0.03***	0.13***
8th year after dissolution								
× treatment × Q1	0.04**	0.14***	− 0.66***	0.00	0.41***	− 0.01	0.21***	0.08***
× treatment × Q2	− 0.01	0.14***	− 0.83***	0.13***	0.48***	0.03*	0.10***	0.09***
× treatment × Q3	− 0.01	0.12***	− 0.89***	0.16***	0.62***	0.01	0.07***	0.03*
× treatment × Q4	− 0.02*	0.13***	− 0.97***	0.22***	0.62***	0.02	0.04***	0.05***
× treatment × Q5	− 0.08***	0.17***	− 1.04***	0.26***	0.56***	0.01	0.02*	0.11***
Unemployment rate								
× Q1	− 0.05***	− 0.02***	− 0.06***	− 0.02***	− 0.01***	0.00**	0.02***	0.02***
× Q2	− 0.03***	− 0.01***	− 0.05***	− 0.01***	− 0.01***	0.01***	0.02***	0.02***
× Q3	− 0.03***	− 0.01***	− 0.04***	− 0.02***	− 0.01***	0.01***	0.01***	0.02***
× Q4	− 0.02***	− 0.01***	− 0.04***	− 0.02***	− 0.01***	0.01***	0.01***	0.02***
× Q5	− 0.02***	− 0.01***	− 0.03***	− 0.02***	− 0.01***	0.01***	0.01***	0.02***
Time × Q dummies	✓	✓	✓	✓	✓	✓	✓	✓
Individual FE	✓	✓	✓	✓	✓	✓	✓	✓
*N* unique persons	96,759	96,759	96,759	96,759	96,759	96,759	96,759	96,759
*N* persons	115,784	115,784	115,784	115,784	115,784	115,784	115,784	115,784
*N* person-years	1,044,702	1,044,702	1,044,702	1,044,702	1,044,702	1,044,702	1,044,702	1,044,702
*R*^2^ overall	0.98	0.91	0.84	0.94	0.66	0.45	0.92	0.86
*R*^2^ within	0.07	0.05	0.33	0.05	0.13	0.02	0.06	0.04
*F* group diff (1st year)	276***	19***	141***	140***	9***	23***	361***	37***
*F* group diff (2nd year)	148***	11***	118***	134***	29***	9***	252***	26***
*F* group diff (3rd year)	92***	8***	106***	111***	40***	6***	184***	23***
*F* group diff (4th year)	66***	4***	95***	103***	48***	1	171***	23***
*F* group diff (5th year)	46***	5***	103***	76***	42***	1	105***	18***
*F* group diff (6th year)	30***	3**	98***	42***	43***	1	70***	12***
*F* group diff (7th year)	22***	2*	80***	36***	32***	1	33***	12***
*F* group diff (8th year)	9***	2*	22***	20***	17***	1	13***	4***

Standard errors accounted for clustering at the individual level. *p < .05, **p < *.*01, ***p < *.*001

**Table 7:** Fixed-effects regressions of changes in income components following union dissolution, using the square root equivalence scale

	Household disp. income	Household composition	First partner earnings	Personal earnings	New partner earnings	Other income	Transfer income	Taxes and contribution
1st year before dissolution								
× treatment × Q1	− 0.01*	0.01	− 0.03***	0.02***	− 0.01***	0.00	0.01	0.01**
× treatment × Q2	0.02***	0.03***	0.01	0.03***	− 0.01***	− 0.01***	0.01***	− 0.01*
× treatment × Q3	0.03***	0.04***	0.03***	0.04***	− 0.01***	− 0.02***	0.01***	− 0.02***
× treatment × Q4	0.03***	0.04***	0.04***	0.05***	− 0.01***	− 0.02***	0.01***	− 0.03***
× treatment × Q5	0.04***	0.04***	0.05***	0.04***	− 0.01***	− 0.01**	0.00*	− 0.04***
1st year after dissolution								
× treatment × Q1	− 0.05***	0.18***	− 0.88***	0.14***	0.06***	0.19***	0.28***	0.15***
× treatment × Q2	− 0.15***	0.17***	− 1.04***	0.22***	0.08***	0.22***	0.12***	0.24***
× treatment × Q3	− 0.16***	0.18***	− 1.06***	0.28***	0.10***	0.22***	0.08***	0.21***
× treatment × Q4	− 0.19***	0.20***	− 1.10***	0.35***	0.09***	0.19***	0.05***	0.22***
× treatment × Q5	− 0.26***	0.23***	− 1.16***	0.38***	0.08***	0.14***	0.03***	0.28***
2nd year after dissolution								
× treatment × Q1	− 0.05***	0.17***	− 0.86***	0.13***	0.13***	0.15***	0.25***	0.15***
× treatment × Q2	− 0.13***	0.16***	− 1.02***	0.21***	0.18***	0.16***	0.11***	0.22***
× treatment × Q3	− 0.13***	0.17***	− 1.03***	0.28***	0.22***	0.16***	0.07***	0.18***
× treatment × Q4	− 0.15***	0.19***	− 1.08***	0.35***	0.23***	0.14***	0.04***	0.17***
× treatment × Q5	− 0.21***	0.21***	− 1.13***	0.37***	0.18***	0.12***	0.03***	0.23***
3rd year after dissolution								
× treatment × Q1	− 0.03***	0.16***	− 0.84***	0.10***	0.23***	0.10***	0.23***	0.13***
× treatment × Q2	− 0.11***	0.15***	− 0.99***	0.19***	0.30***	0.11***	0.10***	0.19***
× treatment × Q3	− 0.09***	0.16***	− 1.01***	0.27***	0.34***	0.12***	0.06***	0.13***
× treatment × Q4	− 0.11***	0.17***	− 1.06***	0.34***	0.37***	0.10***	0.03***	0.11***
× treatment × Q5	− 0.17***	0.20***	− 1.10***	0.35***	0.31***	0.07***	0.02***	0.18***
4th year after dissolution								
× treatment × Q1	− 0.02**	0.16***	− 0.81***	0.09***	0.31***	0.07***	0.21***	0.11***
× treatment × Q2	− 0.08***	0.14***	− 0.96***	0.17***	0.37***	0.08***	0.10***	0.16***
× treatment × Q3	− 0.06***	0.14***	− 0.98***	0.24***	0.44***	0.08***	0.06***	0.09***
× treatment × Q4	− 0.07***	0.15***	− 1.03***	0.32***	0.46***	0.08***	0.03***	0.08***
× treatment × Q5	− 0.15***	0.17***	− 1.09***	0.32***	0.38***	0.07***	0.02***	0.16***
5th year after dissolution								
× treatment × Q1	− 0.01	0.17***	− 0.77***	0.05***	0.36***	0.05***	0.19***	0.10***
× treatment × Q2	− 0.07***	0.13***	− 0.94***	0.16***	0.42***	0.06***	0.09***	0.13***
× treatment × Q3	− 0.05***	0.13***	− 0.95***	0.22***	0.51***	0.06***	0.05***	0.07***
× treatment × Q4	− 0.05***	0.14***	− 1.02***	0.28***	0.54***	0.05***	0.03***	0.07***
× treatment × Q5	− 0.13***	0.16***	− 1.09***	0.29***	0.45***	0.04***	0.02***	0.15***
6th year after dissolution								
× treatment × Q1	− 0.01	0.14***	− 0.75***	0.03	0.39***	0.04**	0.18***	0.10***
× treatment × Q2	− 0.06***	0.12***	− 0.91***	0.14***	0.45***	0.05***	0.10***	0.12***
× treatment × Q3	− 0.04***	0.12***	− 0.93***	0.20***	0.55***	0.03***	0.06***	0.06***
× treatment × Q4	− 0.04***	0.13***	− 1.00***	0.24***	0.60***	0.03***	0.03***	0.06***
× treatment × Q5	− 0.11***	0.16***	− 1.07***	0.28***	0.49***	0.04***	0.02***	0.13***
7th year after dissolution								
× treatment × Q1	− 0.01	0.13***	− 0.70***	0.02	0.39***	0.02	0.15***	0.11***
× treatment × Q2	− 0.05***	0.11***	− 0.88***	0.12***	0.47***	0.04***	0.08***	0.11***
× treatment × Q3	− 0.03***	0.12***	− 0.89***	0.17***	0.57***	0.01	0.06***	0.05***
× treatment × Q4	− 0.04***	0.12***	− 0.97***	0.21***	0.60***	0.02*	0.04***	0.06***
× treatment × Q5	− 0.11***	0.15***	− 1.06***	0.25***	0.51***	0.02*	0.02**	0.14***
8th year after dissolution								
× treatment × Q1	0.00	0.13***	− 0.64***	0.00	0.41***	− 0.03	0.15***	0.10***
× treatment × Q2	− 0.05***	0.12***	− 0.86***	0.10***	0.48***	0.02	0.10***	0.11***
× treatment × Q3	− 0.03**	0.11***	− 0.88***	0.13***	0.60***	0.01	0.07***	0.05***
× treatment × Q4	− 0.03**	0.11***	− 0.96***	0.20***	0.61***	0.02	0.03***	0.06***
× treatment × Q5	− 0.11***	0.16***	− 1.04***	0.21***	0.56***	0.01	0.02*	0.13***
Unemployment rate								
× Q1	− 0.05***	− 0.02***	− 0.06***	− 0.02***	− 0.01***	0.00**	0.02***	0.02***
× Q2	− 0.03***	− 0.01***	− 0.04***	− 0.01***	− 0.01***	0.01***	0.01***	0.02***
× Q3	− 0.03***	− 0.01***	− 0.04***	− 0.02***	− 0.01***	0.01***	0.01***	0.02***
× Q4	− 0.03***	− 0.01***	− 0.03***	− 0.02***	− 0.01***	0.01***	0.01***	0.02***
× Q5	− 0.03***	− 0.01***	− 0.03***	− 0.02***	− 0.01***	0.01***	0.01***	0.02***
Time × Q dummies	✓	✓	✓	✓	✓	✓	✓	✓
Individual FE	✓	✓	✓	✓	✓	✓	✓	✓
*N* unique persons	96,772	96,772	96,772	96,772	96,772	96,772	96,772	96,772
*N* persons	115,765	115,765	115,765	115,765	115,765	115,765	115,765	115,765
*N* person-years	1,044,655	1,044,655	1,044,655	1,044,655	1,044,655	1,044,655	1,044,655	1,044,655
*R*^2^ overall	0.97	0.91	0.84	0.94	0.69	0.46	0.92	0.86
*R*^2^ within	0.07	0.05	0.33	0.04	0.13	0.02	0.05	0.05
*F* group diff (1st year)	165***	26***	107***	132***	7***	23***	261***	35***
*F* group diff (2nd year)	86***	15***	90***	126***	27***	9***	221***	25***
*F* group diff (3rd year)	52***	11***	82***	118***	34***	5***	166***	24***
*F* group diff (4th year)	39***	5***	73***	90***	32***	1	125***	23***
*F* group diff (5th year)	29***	6***	82***	69***	42***	1	73***	18***
*F* group diff (6th year)	19***	4***	72***	39***	41***	1	51***	13***
*F* group diff (7th year)	15***	3**	67***	31***	28***	1	25***	11***
*F* group diff (8th year)	7***	2*	49***	17***	17***	2*	16***	6***

Standard errors accounted for clustering at the individual level. * *p* < .05, ** *p* < *.*01, *** *p* < *.*001

A third analysis concerned the stratifier. The previous sections compared women by pre-dissolution income group, in line with the notion of income convergence. However, sociologists might rather be interested in comparing women by social class, and pre-dissolution income group is an unstable indicator of class as it fluctuates over the life course. Hence, I repeated the analysis by women’s educational attainment. The results showed that income convergence by education level was less pronounced, because higher-educated women compensated more via changes in household composition and personal earnings, and because the educational difference in partner independence was smaller (Table 8 of the Appendix). This is unsurprising because, compared to pre-dissolution household income, a woman’s education level is less closely related to her partner’s earnings and more closely to her own earnings capacity.

**Table 8:** Fixed-effects regressions of changes in income components following union dissolution, by education level

	Household disp. income	Household composition	First partner earnings	Personal earnings	New partner earnings	Other income	Transfer income	Taxes and contribution
1st year before dissolution								
× treatment × ISCED 1–3	0.01*	0.02***	0.00	0.02***	− 0.01***	− 0.01**	0.01**	0.00
× treatment × ISCED 4–5	0.02***	0.03***	0.01	0.04***	− 0.01**	− 0.01**	0.01*	− 0.01*
× treatment × ISCED 6–8	0.02***	0.03***	0.02**	0.05***	− 0.02***	− 0.02***	0.00	− 0.02***
1st year after dissolution								
× treatment × ISCED 1–3	− 0.12***	0.18***	− 0.97***	0.15***	0.06***	0.20***	0.22***	0.23***
× treatment × ISCED 4–5	− 0.14***	0.21***	− 1.12***	0.27***	0.09***	0.25***	0.14***	0.23***
× treatment × ISCED 6–8	− 0.16***	0.28***	− 1.06***	0.40***	0.08***	0.16***	0.06***	0.20***
2nd year after dissolution								
× treatment × ISCED 1–3	− 0.11***	0.18***	− 0.94***	0.13***	0.15***	0.15***	0.20***	0.21***
× treatment × ISCED 4–5	− 0.11***	0.20***	− 1.08***	0.26***	0.21***	0.17***	0.12***	0.20***
× treatment × ISCED 6–8	− 0.11***	0.22***	− 1.05***	0.39***	0.21***	0.14***	0.05***	0.15***
3rd year after dissolution								
× treatment × ISCED 1–3	− 0.08***	0.16***	− 0.90***	0.11***	0.25***	0.10***	0.18***	0.18***
× treatment × ISCED 4–5	− 0.08***	0.19***	− 1.05***	0.24***	0.34***	0.13***	0.11***	0.16***
× treatment × ISCED 6–8	− 0.07***	0.20***	− 1.04***	0.38***	0.34***	0.11***	0.04***	0.09***
4th year after dissolution								
× treatment × ISCED 1–3	− 0.07***	0.15***	− 0.88***	0.09***	0.31***	0.08***	0.17***	0.15***
× treatment × ISCED 4–5	− 0.06***	0.17***	− 1.03***	0.21***	0.43***	0.09***	0.11***	0.13***
× treatment × ISCED 6–8	− 0.04***	0.18***	− 1.01***	0.34***	0.46***	0.07***	0.04***	0.06***
5th year after dissolution								
× treatment × ISCED 1–3	− 0.06***	0.14***	− 0.84***	0.07***	0.36***	0.06***	0.16***	0.14***
× treatment × ISCED 4–5	− 0.03***	0.16***	− 0.99***	0.20***	0.49***	0.07***	0.10***	0.10***
× treatment × ISCED 6–8	− 0.03***	0.17***	− 1.00***	0.30***	0.53***	0.05***	0.05***	0.05***
6th year after dissolution								
× treatment × ISCED 1–3	− 0.05***	0.14***	− 0.80***	0.06***	0.39***	0.04***	0.14***	0.12***
× treatment × ISCED 4–5	− 0.04***	0.15***	− 0.97***	0.17***	0.54***	0.03**	0.09***	0.09***
× treatment × ISCED 6–8	− 0.01	0.06	− 0.99***	0.25***	0.59***	0.03**	0.05***	0.05**
7th year after dissolution								
× treatment × ISCED 1–3	− 0.05***	0.12***	− 0.76***	0.03**	0.39***	0.03***	0.14***	0.13***
× treatment × ISCED 4–5	− 0.04***	0.14***	− 0.93***	0.13***	0.53***	0.03	0.10***	0.11***
× treatment × ISCED 6–8	− 0.02*	0.15***	− 0.97***	0.23***	0.61***	0.02*	0.05***	0.05***
8th year after dissolution								
× treatment × ISCED 1–3	− 0.04***	0.13***	− 0.72***	0.03*	0.40***	− 0.01	0.13***	0.12***
× treatment × ISCED 4–5	− 0.04**	0.13***	− 0.85***	0.10***	0.50***	0.01	0.08***	0.11***
× treatment × ISCED 6–8	− 0.02	0.15***	− 0.98***	0.19***	0.64***	0.03*	0.04***	0.05***
Unemployment rate								
× ISCED 1–3	− 0.04***	− 0.02***	− 0.05***	− 0.02***	− 0.01***	0.00***	0.02***	0.02***
× ISCED 4–5	− 0.03***	− 0.01***	− 0.05***	− 0.02***	− 0.01***	0.01***	0.02***	0.02***
× ISCED 6–8	− 0.03***	− 0.01*	− 0.04***	− 0.03***	− 0.01***	0.01***	0.01***	0.03***
Time x ISCED dummies	✓	✓	✓	✓	✓	✓	✓	✓
Individual FE	✓	✓	✓	✓	✓	✓	✓	✓
*N* unique persons	72,048	72,048	72,048	72,048	72,048	72,048	72,048	72,048
*N* persons	87,325	87,325	87,325	87,325	87,325	87,325	87,325	87,325
*N* person-years	798,463	798,463	798,463	798,463	798,463	798,463	798,463	798,463
*R*^2^ overall	0.98	0.78	0.87	0.94	0.67	0.48	0.92	0.88
*R*^2^ within	0.03	0.00	0.29	0.04	0.12	0.02	0.05	0.03
*F* group diff (1st year)	17***	9***	59***	297***	5***	24***	310***	7***
*F* group diff (2nd year)	1	12***	60***	283***	25***	5***	266***	29***
*F* group diff (3rd year)	2*	9***	76***	255***	50***	3**	211***	48***
*F* group diff (4th year)	6***	5***	67***	211***	69***	2*	149***	48***
*F* group diff (5th year)	8***	4***	75***	154***	100***	1	83***	42***
*F* group diff (6th year)	8***	1	87***	54***	102***	1	40***	9***
*F* group diff (7th year)	4***	2*	78***	53***	75***	0	25***	18***
*F* group diff (8th year)	2*	1	73***	26***	44***	2*	12***	8***

Educational attainment categorized using the International Standard Classification of Education (ISCED). The number of observations is smaller than in the main analysis because education certificates were incompletely registered before 2003. Standard errors accounted for clustering at the individual level. *p < .05, **p < *.*01, ***p < *.*001

## Discussion

Union dissolution is a critical event for women’s living standards. Surprisingly, previous research has found that women from high-income unions lose relatively much, whereas women from low-income unions witness little change (Bonnet et al., 2021; Brewer & Nandi, 2014; Duncan & Hoffman, 1985; Fisher & Low, 2016; Jarvis & Jenkins, 1999; Rowe & Lown, 1990; Uunk, 2004; Weiss, 1984; Weitzman, 1981). This has raised the question of why socioeconomic differences in women’s living standards “converge” after dissolution.

Hence, this study examined the convergence in women’s living standards following union dissolution. It proposed two mechanisms explain the convergence: compensation and partner independence. Using administrative data from the Netherlands, I estimated the dissolution penalty by pre-dissolution income group. A decomposition analysis showed that the convergence in living standards was not driven by compensation. Women from lower-income unions had a compensatory advantage regarding transfer income, neither an advantage nor disadvantage regarding household composition and new partner earnings, and a disadvantage regarding personal earnings and tax payments. Instead, convergence was driven by the partner independence mechanism. Women in lower-income unions were financially more independent of their partner’s earnings because they relied more on tax-benefit income, so they stood to lose less from dissolution.

These results demonstrate a dual role of the welfare state. To an extent, the state reduces the negative consequences of union dissolution through post-dissolution compensation. Reductions in taxes mitigate the losses of women from high-income unions, while increases in transfers mitigate the losses of women from low-income unions. However, the prime influence of the state passes through pre-dissolution partner independence. Greater reliance on public transfers reduces the portion of partner-tied income that is lost upon dissolution. Attention to this dual role is important. Many studies have found a smaller dissolution penalty when looking at net income than at market income, yet noticed that this smaller penalty could not be fully explained by post-dissolution compensation (Bonnet et al., 2021; Bratberg & Tjøtta, 2008; Fisher & Low, 2016; Jarvis & Jenkins, 1999; Tach & Eads, 2015). The current study shows that the missing link here is that the welfare state also influences how much partner income can be lost in the first place.

This leads to an interesting conclusion about the concept of independence. Women’s financial independence is usually conceptualized with reference to the market: women who earn more similarly to or who out-earn their partners are said to be more independent. The current study shows that independence can also be facilitated by the state: a large share of household income from public transfers enables women to maintain a standard of living relatively independently of a partner. What we call welfare dependency, then, could actually grant women the “autonomy” (Orloff, 1993) to decide on the continuation of their relationships. Nevertheless, this autonomy is limited, as the state increasingly subjects welfare claimants to surveillance (Gaffney & Millar, 2020).

An ensuing question is what welfare policies contribute to the financial independence of women from low-income unions. The Netherlands features several means-tested schemes, notably social assistance, child assistance, and childcare benefits. The income thresholds of these schemes are generous enough to entitle most households in the lower quintiles to receiving them (Avram et al., 2014), protecting couples in the lower quintiles against the consequences of union dissolution. At the same time, the very fact that most benefits are means tested, together with expensive childcare services and a tax credit for informal caregivers, discourages women in the lower quintiles from working full time (Evertsson et al., 2009) and limits earnings increases after union dissolution. A full understanding of the policies that influence compensation and independence, and how they work in different contexts, would therefore require further study. One possibility is the simulation study, in which incomes are simulated under existing and hypothetical policies using tools like Taxsim or Euromod (Popova & Navicke, 2019). In the European context, a recent adaptation of the German Socio-Economic Panel for usage with Euromod provides a promising avenue for doing so (Bartels et al., 2021).

What do the results mean for social inequality? On the one hand, they paint a positive picture. Redistribution by the welfare state seems to protect women with a low socioeconomic status against the consequences of union dissolution. On the other hand, women with an intermediary or high socioeconomic status incur large losses. The convergence studied here could therefore be considered “perverse” or “convergence to the bottom” (Bloome, 2017). In addition, the focus on incomes tells only part of the story. Women with a lower status are less affected by dissolution on average, but this average masks upward and downward mobility. Other studies have shown that union dissolution increases women’s risk of moving to the extremes of the income distribution (Ananat & Michaels, 2008). Indeed, the share of women who fall below the poverty line is larger in the lower socioeconomic strata (Hogendoorn et al., 2020) because minimum income provisions are insufficient for lifting people above the poverty line (Nelson, 2013). In other words, the protection of women in lower strata is noticeable yet incomplete.

On a related note, the results have implications for intergenerational inequality. It is well known that parental separation lowers children’s educational attainment (for a review, see Bernardi & Boertien, 2017). This applies especially to children of highly educated mothers, who experience larger losses of household income (Bernardi & Boertien, 2016). The current study shows that this is because highly educated mothers are financially more dependent on fathers’ earnings. This suggests that universal (as opposed to means-tested) policies will reduce the parental separation penalty especially for children of highly educated mothers. If this is true, however, then universal policies will strengthen the intergenerational transmission of education across family structures: parental separation will no longer put children from different parental backgrounds on the same level. Policies could thus invoke a trade-off between children’s attainment and intergenerational equality.

Notwithstanding these findings, there are some limitations to this study. One limitation regards the measurement of living standards. My measure did not include the value of unpaid care and housework, while women spend significant amounts of time on unpaid work (Gupta et al., 2021; Mansour & McKinnish, 2014). Preliminary work suggests that including unpaid work would hardly change the dissolution penalty (Bratberg & Tjøtta, 2008), but more work is needed. Another limitation is that income is distinct from expenditure. Expenses related to union dissolution can be considerable and include divorce litigation, the repurchase of household goods, and double mortgage or rent payments. A broader view on women’s living standards could benefit from the measurement of such expenses, which are not available in administrative registers. Furthermore, the data did not allow me to assign tax payments to individual household members, because taxes include a small component levied at the household level. Individual assignment would not change the statistical results, but it could change their interpretation: what currently appears as a reduction in tax payments that benefits separating women may represent the cessation of taxes levied on the partner’s earnings. This would mean that women from higher-income unions do not enjoy a small compensatory advantage due to progressive taxation and that their financial dependence on the partner is somewhat less sizeable. Lastly, this study relies on decomposition analysis. The decomposition untangled the relative importance of household composition and income sources for the dissolution penalty. In reality, of course, these components interact. Increases in earnings reduce entitlements to transfers, households composed of fewer children become entitled to fewer child benefits, and so forth. A more comprehensive design might be able to incorporate these interactions, and the current study represents just a first step in that direction.

To conclude, this study examined the compensation and partner independence mechanisms for the event of union dissolution. These mechanisms easily extend to other life events. Examples include labor market events, such as job loss, sickness, disability, or retirement, and demographic events, such as childbirth, home leaving, or bereavement. The consequences of those events, too, depend on the contributions of other household members and their embedding in institutional arrangements. Future work may examine these to shed more light on the nexus between life events, social policy, and stratification.

## Appendix

See Tables 3, 4, 5, 6, 7 and 8.

## Footnotes

1 There are three types of fiscal unity in the Netherlands. Non-partners are taxed individually, cannot share property tax allowances, and only their own means are tested when requesting benefits; benefit partners are taxed individually, cannot share property tax allowances, but are jointly means tested; fiscal partners are taxed individually, but may share property tax allowances and are jointly means-tested. Contractual cohabitants, registered partners, and married spouses are automatically fiscal partners. Non-contractual cohabitants are under most conditions automatically fiscal partners and, even those who are not, automatically qualified as benefit partners between 2005 and 2013 if they cohabited for more than six months. Hence, in our data, nearly all couples are subject to joint means tests.

2 I drew on the union file as updated in spring 2021. The file retroactively identified couples if they had lived together at two addresses or if they had met any of the other conditions between 2003 and 2021. To illustrate, at least 65% of Dutch couples move to a new address within five years after union formation (Feijten & Mulder, 2002), 87% of couples have registered their relationship (Poortman & Mills, 2012), 70% of couples have children (Statistics Netherlands, 2021), and at least 57% of households receive couple-level benefits (Bos, 2016).

3 In rare cases, women had formed multiple unions between 2003 and 2005 (0.03% two, < 0.01% three unions). When this occurred, I selected the first union for analysis.

4 Incomplete observations were caused mostly by missing tax returns. Of all incomplete observations, 84% missed an income variable, 4% another variable, and 12% missed both. This was because tax returns were measured “in real time”: people who filed their taxes incorrectly received a corrected tax bill one year later, which was reflected in the data with a missing score in the first year and the corrected score in the next. Demographic differences between incomplete and complete person-years were negligible.

5 In households with one adult, the equivalence factors were 1.00 (zero children), 1.33 (one child), 1.51 (two), 1.76 (three), 1.95 (four). In households with two adults, equivalence factors were 1.37 (zero), 1.67 (one), 1.88 (two), 2.06 (three). In all other cases, the equivalence scale was $\sqrt{1*Adults+0.8*Children}$.

6 I experimented extensively with alternative model specifications. Eventually, time since union dissolution dummies were interacted with the treatment indicator only in the first year before dissolution and all years after dissolution. Interactions in further years before dissolution were virtually non-existent due to matching, were not of theoretical interest, did not improve the model fit, and substantially reduced model parsimony. Complete elimination of any interactions in further years before dissolution could be achieved by also matching on characteristics in those years, but doing so would have resulted in many persons being dropped due to missing observations and would have forced me to only consider the select group of women whose unions survived multiple years.
